# Resposta à carta ao editor sobre “Descrição das deformidades do quadril em pacientes com síndrome congênita do Zika vírus aos 5 anos de idade: Um estudo transversal”

**DOI:** 10.1055/s-0045-1814119

**Published:** 2025-12-30

**Authors:** Herison Franklin Viana de Oliveira, Francisco Robson Queiroz Rego, Brauner de Souza Cavalcanti, Ítalo Rodrigues Bacellar, Thais Araújo Barbosa, Epitácio Leite Rolim Filho

**Affiliations:** 1 Departamento de Cirurgia, Centro de Ciências Médicas, Universidade Federal de Pernambuco, Recife, PE, Brasil; 2 Departamento de Ortopedia e Traumatologia, Hospital Getúlio Vargas, Recife, PE, Brasil; 3 Departamento de Ortopedia e Traumatologia, Centro de Ciências Médicas, Universidade Federal de Pernambuco, Recife, PE, Brasil

Agradecemos a observação pertinente quanto ao risco de viés de seleção. Reconhecemos que, por se tratar de um estudo transversal retrospectivo, essa limitação metodológica não pode ser completamente eliminada. No entanto, procuramos reduzir ao máximo esse viés ao incluir todas as crianças com síndrome congênita do vírus Zika que compareceram consecutivamente ao ambulatório durante o período analisado, desde que preenchessem os critérios de inclusão previamente definidos. Dessa forma, não houve escolha arbitrária dos casos, mas sim a utilização de uma amostra representativa da população atendida em um serviço de referência. Consideramos que essa estratégia contribuiu para minimizar de maneira relevante a possibilidade de distorções nos resultados, ainda que não elimine completamente a limitação inerente ao desenho retrospectivo.

Concordamos com a observação de que o tamanho da amostra (62 pacientes) pode limitar análises estatísticas mais complexas, sobretudo quando os dados são estratificados por níveis do sistema de classificação da função motora grossa (SCFMG), o que naturalmente reduz o número de indivíduos em cada subgrupo e, consequentemente, a precisão e o poder estatístico. Reconhecemos também que a ausência de intervalos de confiança e de controle para variáveis de confusão, como comorbidades ou fatores nutricionais, constitui uma limitação do presente estudo. Todavia, ressaltamos que seu objetivo foi fornecer uma descrição inicial das alterações estruturais do quadril em uma população específica de crianças com síndrome congênita do vírus Zika, ainda pouco explorada na literatura. Destacamos que a superação dessas limitações demanda estudos multicêntricos, com maior número de participantes e metodologia prospectiva, o que certamente contribuirá para análises mais robustas e melhor controle de variáveis de confusão.

Entendemos que a ausência de detalhamento metodológico quanto ao controle das covariáveis pode, de fato, ter limitado a interpretação dos resultados. De todo modo, ressaltamos que, em nossa análise, as variáveis índice de Reimers (IR), índice acetabular (IA) e ângulo colodiafisário (ACD) foram pareadas entre os pacientes submetidos à tenotomia e aqueles que não realizaram o procedimento, buscando diferenças estatísticas. 1 Reconhecemos, que este é um ponto relevante a ser aprimorado em futuras versões e em estudos subsequentes com maior número de casos.

Agradecemos as considerações apresentadas, especialmente no que se refere às questões levantadas para fomentar a discussão acadêmica. Reconhecemos que tais indagações são extremamente pertinentes e enriquecem a reflexão sobre os achados de nosso estudo. Embora não tenham sido diretamente exploradas nesta análise, consideramos que esses pontos representam caminhos relevantes para investigações futuras e poderão subsidiar o desenvolvimento de novos trabalhos com delineamentos mais específicos.

São valiosas as sugestões quanto às possíveis direções para estudos futuros. Consideramos extremamente pertinentes as propostas de delineamentos longitudinais, amostras ampliadas e multicêntricas, bem como a incorporação de novas tecnologias, como modelagem tridimensional (3D) e inteligência artificial, para aprofundar a compreensão das alterações estruturais e funcionais do quadril em crianças com síndrome congênita do Zika. Certamente, tais abordagens representam caminhos promissores para gerar evidências mais robustas e subsidiar estratégias de cuidado individualizado para essa população.
